# Late-Onset Normotensive Thrombotic Microangiopathy and Pyoderma Gangrenosum Following Nine Years of Sunitinib Therapy: A Case Report

**DOI:** 10.1016/j.xkme.2026.101298

**Published:** 2026-02-13

**Authors:** Ryosuke Saiki, Kan Katayama, Makiko Yajima, Takeshi Sasaki, Yuri Oue, Tomohiro Murata, Kaoru Dohi

**Affiliations:** 1 Department of Cardiology and Nephrology, Mie University Graduate School of Medicine, Tsu, Japan; 2 Department of Dermatology, Mie University Graduate School of Medicine, Tsu, Mie, Japan; 3 The Department of Urology, Nephro-Urologic Surgery and Andrology, Mie University Graduate School of Medicine, Tsu, Mie, Japan

**Keywords:** Concurrent, late-onset, pyoderma gangrenosum, sunitinib, thrombotic microangiopathy (TMA)

## Abstract

Sunitinib, a tyrosine kinase inhibitor used for metastatic renal cell carcinoma, is associated with various adverse effects. We present the case of a 60-year-old woman who developed biopsy-proven thrombotic microangiopathy and concurrent pyoderma gangrenosum after 9 years of sunitinib therapy. This case is unusual due to the extremely delayed onset of both rare toxicities. Furthermore, the thrombotic microangiopathy presented without hypertension, a typical preceding sign, which made the diagnosis challenging. The patient initially presented with a painful leg ulcer, diagnosed as pyoderma gangrenosum, and was subsequently found to have significant proteinuria and edema. A kidney biopsy confirmed thrombotic microangiopathy. Upon discontinuation of sunitinib, both the proteinuria and the pyoderma gangrenosum lesions improved significantly, confirming a causal relationship. This case represents the longest reported latency period for both sunitinib-induced thrombotic microangiopathy and pyoderma gangrenosum. It underscores the critical need for sustained clinical vigilance for severe, late-onset adverse events at any point throughout long-term sunitinib treatment and demonstrates that clinicians cannot rely solely on hypertension as a predictive marker for thrombotic microangiopathy.

Sunitinib is a molecularly targeted therapy used in the treatment of metastatic renal cell carcinoma (RCC).^1^ This multitargeted tyrosine kinase inhibitor inhibits vascular endothelial growth factor receptor 2, stem cell factor receptor (c-kit), fetal liver tyrosine kinase receptor 3, and platelet-derived growth factor receptors A and B,^2^ thereby suppressing tumor angiogenesis and inhibiting tumor growth and metastasis.^3^ The main adverse effects of sunitinib include diarrhea, hypertension, dermatologic toxicities, hypothyroidism, fatigue, and nausea.^3^ Although sunitinib has proven efficacy in treating renal cell carcinoma, its broad receptor inhibition profile can lead to various adverse effects ranging from common toxicities to rare but serious complications.

Thrombotic microangiopathy (TMA) typically presents with microangiopathic hemolytic anemia, thrombocytopenia, and organ injury.^4^ A renal-limited form of TMA has also been reported, and it often lacks the hematologic abnormalities (such as thrombocytopenia and hemolytic anemia) seen in typical TMA.^5^ Sunitinib-induced TMA is considered to be caused by dose- and duration-dependent toxicity.^4^ Renal TMA associated with anti-vascular endothelial growth factor (VEGF) agents typically develops with a mean onset ∼3 months after treatment initiation.^6^ Usually, hypertension precedes sunitinib-induced TMA,^7^ making it a useful diagnostic marker.

Pyoderma gangrenosum (PG), a neutrophilic dermatosis characterized by painful, ulcerative lesions with a distinct border, is most commonly associated with underlying diseases such as inflammatory bowel disease, rheumatoid arthritis, myelodysplastic syndrome, or hematologic malignancies.^2^^,^^8^ However, PG is also a rare adverse event associated with sunitinib.^8^ A systematic review identified only 15 cases of tyrosine kinase inhibitor-associated PG, with a median time to onset of 5.6 months (range: 6 days to 18 months) from drug initiation.^8^

In this report, we describe a patient who developed normotensive, biopsy-proven TMA and concurrent PG after 9 years of sunitinib therapy. By presenting this complex case with an unusually late presentation, we aim to expand the current understanding of sunitinib toxicity and emphasize the importance of ongoing vigilance for atypical adverse events during long-term tyrosine kinase inhibitor treatment.^8^

## Case Report

A 60-year-old woman was referred to our nephrology service for evaluation of worsening edema and proteinuria. Fourteen years prior, she had undergone a laparoscopic left nephrectomy for clear cell renal cell carcinoma (pT1aN0M0). Nine years prior, she developed recurrent disease with masses in the right thoracic wall and right iliac muscle, for which she was started on sunitinib. Prior to the initiation of sunitinib, her systolic blood pressure was consistently ∼120 mm Hg. After treatment began, it increased to the 130s, leading to the prescription of an angiotensin II receptor blocker. A year after she started sunitinib, a calcium channel blocker was added, and her blood pressure subsequently stabilized. Her home systolic readings were consistently 110-130 mm Hg from that point on until her visit. Three years prior, although she achieved a complete response, a 3-month pause in treatment led to the enlargement of lung metastases, necessitating the resumption of sunitinib at a dose of 25 mg on a one-week-on, one-week-off schedule. Although 1+ proteinuria had been noted on qualitative urinalysis for the past year, the patient experienced no subjective symptoms, such as edema. Her medical history was also significant for hypothyroidism, diagnosed before sunitinib initiation and managed with levothyroxine. She had no other significant comorbid conditions.

**Figure 1:**
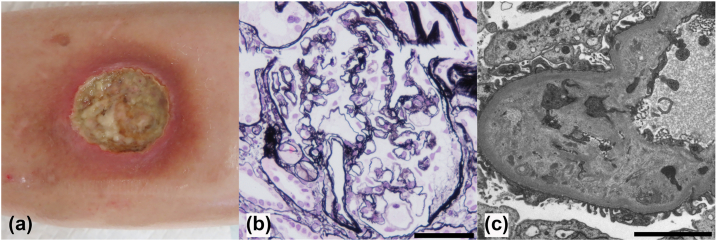
Skin and kidney findings. (a) Painful, well-demarcated ulcer on the patient's left lower leg. (b) Periodic acid methenamine silver staining shows diffuse duplication of the basement membrane. Scale bar = 50 μm. (c) Electron microscopy reveals marked expansion of the subendothelial space. Scale bar = 5 μm.

One week prior to her nephrology referral, she developed a painful, well-demarcated ulcer on her left lower leg (Fig 1a), for which a skin biopsy was performed. The skin biopsy revealed infiltration of white blood cells, including neutrophils and lymphocytes, in the dermis, but no evidence of malignancy was found. Based on the macroscopic appearance of the skin lesion and histological findings, she was diagnosed with PG. At the time of skin biopsy, lower extremity edema was already present, and subsequent detection of proteinuria (4+ on urinalysis) prompted referral to nephrology for further evaluation. On presentation, her blood pressure was 125/78 mm Hg. Physical examination revealed pitting edema in the lower extremities and the previously diagnosed PG lesion on her left leg.

Laboratory data are presented in Table 1. Initial laboratory investigations showed a serum creatinine of 1.10 mg/dL (estimated glomerular filtration rate 39.9 mL/min/1.73 m^2^), a platelet count of 156,000/μL, and urinary protein of 3.22 g/g creatinine. The 50% hemolytic complement activity was normal. As TMA was not strongly suspected at first, a peripheral blood smear was not examined, nor was ADAMTS13 (von Willebrand factor protease) activity measured.

**Table 1:** Laboratory Data Before Kidney Biopsy.

Urinary Examination (Reference Range)	Value	Blood Chemistry (Reference Range)	Value
pH (4.5-7.5)	7	Total protein, g/dL (6.6-8.1)	5.1
Protein, g/g Cr	3.22	Albumin, g/dL (4.1-5.1)	2.4
Occult blood	Negative	BUN, mg/dL (8-20)	15.2
NAG, U/L (0.0-11.5)	7.6	Cr, mg/dL (0.46-0.79)	1.1
RBC, per high power field	<1	eGFR, mL/min/1.73 m^2^	39.9
Bence-Jones protein	Negative	Uric acid, mg/dL (2.6-5.5)	3.5
		Sodium, mEq/L (138-145)	141
**Complete blood count**		Potassium, mEq/L (3.6-4.8)	4.6
WBC, per μL (3,300-8,600)	3,020	Chloride, mEq/L (101-108)	103
RBC, ×10^4^/μL (435-555)	364	Calcium, mg/dL (8.8-10.1)	8.5
Hemoglobin, g/dL (13.7-16.8)	12.2	AST, U/L (13-30)	28
Platelets, ×10^4^/μL (15.8-34.8)	15.6	ALT, U/L (7-23)	17
		LDH, U/L (124-222)	302
**Serology**		CPK, U/L (41-153)	85
Antinuclear antibody	<1:40	CRP, mg/dL (0-0.14)	2.18
MPO-ANCA, IU/mL (0-3.5)	<0.2		
PR3-ANCA, IU/mL (0-2.0)	<0.6	IgG, mg/dL (861-1,747)	506
Anti-dsDNA, IU/mL (0-10.0)	0.8	IgA, mg/dL (93-393)	85
		IgM, mg/dL (33-183)	247
		CH50, U/mL (31.6-57.6)	53.3

Abbreviations: ALT, alanine aminotransaminase; Anti-dsDNA, anti-double stranded DNA antibody; AST, aspartate aminotransaminase; BUN, blood urea nitrogen; CH50, 50% hemolytic complement activity; CPK, creatine phosphokinase; Cr, creatinine; CRP, C-reactive protein; eGFR, estimated glomerular filtration rate; Ig; immunoglobulin, LDH, lactate dehydrogenase; MPO-ANCA, myeloperoxidase antineutrophil cytoplasmic antibody; NAG, N-acetyl-β-D-glucosaminidase; PR3-ANCA, proteinase-3-anti-neutrophil cytoplasmic antibody; RBC, red blood cells; WBC, white blood cells.

**Figure 2:**
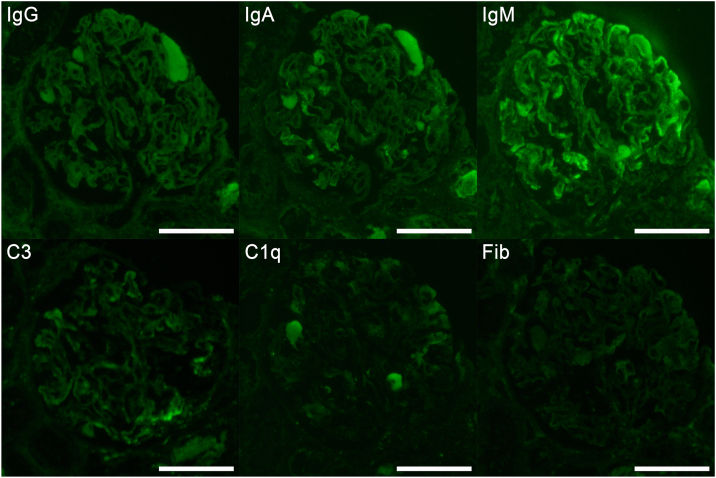
Immunofluorescence study. Immunofluorescent staining shows that IgM is positive in a granular pattern along the capillary walls, and IgA and C3 are segmentally positive in a granular pattern along the capillary walls. Scale bars = 100 μm. Fib, fibrinogen; Ig, immunoglobulin.

A kidney biopsy revealed 2 glomeruli with global sclerosis and 1 with mesangial cell proliferation, among 32 in total. A noteworthy finding was diffuse duplication of the basement membrane (Fig 1b). Electron microscopy revealed marked expansion of the subendothelial space (Fig 1c). Immunofluorescent staining showed that IgM was positive in a granular pattern along the capillary walls and that IgA and C3 were segmentally positive in a granular pattern along the capillary walls (Fig 2). Based on these results, the patient was diagnosed with renal TMA.

Given the biopsy findings, sunitinib was discontinued. For the PG, no systemic medications were administered; treatment consisted of initial topical corticosteroid application followed by continuous topical application of silver sulfadiazine and alprostadil alfadex. These interventions resulted in marked improvement of both proteinuria and the PG lesions. Follow-up laboratory tests 2 months after sunitinib discontinuation demonstrated improvement in renal function with serum creatinine of 0.92 mg/dL, albumin of 3.5 g/dL, and urinary protein of 0.29 g/g creatinine. Her edema also resolved completely. With sunitinib permanently discontinued, the patient is currently scheduled to begin treatment with nivolumab.

## Discussion

This report describes an exceptional case of biopsy-proven, normotensive TMA and concurrent PG developing after an unprecedented 9 years of sunitinib therapy. The case is remarkable for the extremely delayed onset of both rare toxicities, the atypical presentation of TMA without hypertension, and their simultaneous occurrence. The successful resolution of both conditions following drug discontinuation confirms a causal relationship and expands our understanding of sunitinib’s long-term safety profile.

The presentation of TMA was particularly instructive due to its extremely delayed onset and the absence of hypertension, a typical preceding sign of sunitinib-induced TMA.^7^ The mean onset period for renal TMA associated with anti-VEGF agents is approximately 3 months,^6^ making our case’s 9-year latency period remarkably prolonged. The previously reported latest case occurred at 8 years,^9^ and to our knowledge, this represents the longest reported latency period for TMA associated with anti-VEGF agents. This delayed onset is likely the result of cumulative endothelial injury from long-term sunitinib administration. The kidney is particularly susceptible to this mechanism, as glomerular capillary endothelial cells preferentially express VEGF receptors, and VEGF produced by podocytes is essential for the maintenance of the integrity of these cells.^7^ Our patient was treated with a relatively low-dose regimen of 25 mg on a one-week-on, one-week-off schedule. Although this dosing regimen may have avoided acute severe toxicity, it potentially allowed for the accumulation of subthreshold vascular endothelial damage over an extended period. Furthermore, sunitinib has been reported to cause hypertension in a concentration-dependent manner.^10^ The absence of hypertension in our case may be explained by the low-dose, intermittent dosing regimen not reaching the threshold for systemic blood pressure elevation, while gradually accumulated damage may have acted in a renal-limited manner.

Similar to TMA, the development of PG in our case represents an exceptionally delayed onset. A systematic review reported a median time from drug initiation to PG development of 5.6 months, with the longest case occurring at 18 months.^7^ Our subsequent literature search confirmed that this 9-year latency period is the longest documented for PG associated with anti-VEGF agents, far exceeding the next longest report of 3 years.^11^ A possible mechanism for sunitinib-associated PG involves keratinocyte alteration through c-kit inhibition and/or VEGF receptor inhibition, leading to impairment in endothelial cell maintenance.^2^ The parallel, delayed emergence of both TMA and PG in our patient strongly suggests a shared pathophysiology rooted in cumulative, organ-specific toxicity developing over an extended period.

This case offers critical clinical implications for managing patients on long-term sunitinib. It underscores that sustained vigilance is essential throughout the entire treatment course, as severe adverse events can manifest even after years of stable therapy. Clinicians must also recognize that hypertension is not a reliable predictive marker for TMA. Given the duplication of the glomerular basement membrane, which is suggestive of chronic TMA, and a 1-year history of 1+ proteinuria on dipstick urinalysis, the patient may have been presenting with TMA for a year prior to diagnosis. The absence of hypertension should not preclude the diagnosis in patients presenting with proteinuria or renal decline. Consequently, a kidney biopsy becomes an indispensable tool when TMA cannot be diagnosed based solely on clinical and laboratory findings. In our case as well, histopathological examination was essential for confirming TMA and guiding appropriate management.

In conclusion, prolonged tolerance of anti-VEGF agents does not ensure safety. Serious late-onset adverse events—including renal TMA and PG—may arise at any time during long-term sunitinib treatment. Clinicians should maintain ongoing surveillance throughout the course of therapy.
